# Tau Protein Mediates APP Intracellular Domain (AICD)-Induced Alzheimer’s-Like Pathological Features in Mice

**DOI:** 10.1371/journal.pone.0159435

**Published:** 2016-07-26

**Authors:** Kaushik Ghosal, Qingyuan Fan, Hana N. Dawson, Sanjay W. Pimplikar

**Affiliations:** 1 Department of Neurosciences, Lerner Research Institute, Cleveland Clinic, Cleveland, Ohio, 44195, United States of America; 2 Department of Neurology, Duke University, Durham, North Carolina, 27710, United States of America; Nathan Kline Institute and New York University Langone Medical Center, UNITED STATES

## Abstract

Amyloid precursor protein (APP) is cleaved by gamma-secretase to simultaneously generate amyloid beta (Aβ) and APP Intracellular Domain (AICD) peptides. Aβ plays a pivotal role in Alzheimer’s disease (AD) pathogenesis but recent studies suggest that amyloid-independent mechanisms also contribute to the disease. We previously showed that AICD transgenic mice (AICD-Tg) exhibit AD-like features such as tau pathology, aberrant neuronal activity, memory deficits and neurodegeneration in an age-dependent manner. Since AD is a tauopathy and tau has been shown to mediate Aβ–induced toxicity, we examined the role of tau in AICD-induced pathological features. We report that ablating endogenous tau protects AICD-Tg mice from deficits in adult neurogenesis, seizure severity, short-term memory deficits and neurodegeneration. Deletion of tau restored abnormal phosphorylation of NMDA receptors, which is likely to underlie hyperexcitability and associated excitotoxicity in AICD-Tg mice. Conversely, overexpression of wild-type human tau aggravated receptor phosphorylation, impaired adult neurogenesis, memory deficits and neurodegeneration. Our findings show that tau is essential for mediating the deleterious effects of AICD. Since tau also mediates Aβ-induced toxic effects, our findings suggest that tau is a common downstream factor in both amyloid-dependent and–independent pathogenic mechanisms and therefore could be a more effective drug target for therapeutic intervention in AD.

## Introduction

Alzheimer’s disease (AD) is a progressive neurodegenerative disease characterized by two hallmark pathologies: neurofibrillary tangles (NFTs) consisting of microtubule-associated protein tau and senile plaques made up of amyloid-β (Aβ) peptides [1]. AD exhibits neurological symptoms that begin with loss of short-term memory and deteriorate into total loss of cognition and executive functions [2, 3]. A significant number of AD patients also show alterations in electroencephalograms (EEG) as well as silent seizures [4–6]. Although, Aβ peptides have been shown to play a pivotal role in AD pathology, there is a growing recognition that AD is a highly complex, multifactorial disorder and that non-Aβ factors also contribute to AD pathogenesis [7–10].

Tau is a neuronal-specific protein that binds and stabilizes microtubules in axons. Hyperphosphorylated tau is unable to bind microtubules and aggregates to form paired helical filaments (PHF), the major constituent of NFTs. In addition to the cytoskeletal binding functions, tau plays prominent roles in modulating several signaling pathways [11]. In AD and other tauopathies such as frontotemporal dementia, tau becomes hyperphosphorylated and accumulates in somato-dendritic compartments. In addition to phosphorylation, tau can undergo a variety of post-translational modifications such as acetylation, glycation, O-GlcNAcylation and ubiquitination (reviewed in [11]). Accumulating evidence suggests that in AD, tau mediates Aβ-induced pathogenic effects and that reducing tau protects against the deleterious effects of Aβ *in vitro* and in mouse models of AD [12]. However, the exact mechanism by which tau mediates Aβ-induced toxicity remains unknown.

APP Intracellular Domain (AICD) is released from APP by the presenilin-mediated cleavage that also causes extracellular release of Aβ. Numerous *in vitro* studies have shown that AICD regulates gene expression [13], alters cell-signaling pathways and causes deleterious effects (reviewed in [14, 15]). We previously showed that AICD-overexpressing transgenic mice (AICD-Tg) recapitulate AD-like features such as hyperphosphorylation of tau, non-convulsive seizures/aberrant EEGs, neural circuit re-organization, impaired memory and neurodegeneration in an age-dependent fashion [16–19]. Interestingly, aberrant phosphorylation of tau is one of the earliest pathologies observed in AICD-Tg mice, raising the possibility that abnormal tau could contribute to subsequent pathological features. Since tau has been shown to mediate the toxic effects of Aβ both *in vitro* and *in vivo* [20, 21], we investigated whether tau was also involved in AICD-induced neurotoxicity. We report that AD-like pathologies observed in AICD-Tg mice were not observed in mice that lacked tau protein, whereas AICD-Tg mice overexpressing human tau (hTau) showed exacerbated pathologies.

## Methods

### Animals

AICD transgenic mice co-expressing AICD and Fe65 (FeCγ25 line) under the control of the CaMKIIα promoter were described previously [16, 19]. AICD-Tg mice were crossed with tau-deficient mice [22] to produce offspring that were AICD;Tau^-/-^. To evaluate the effect of tau overexpression, hTau mice expressing all six of the human tau protein isoforms were used [22]. hTau mice were crossed to AICD transgenic mice to create AICD;hTau, hTau, AICD and WT littermates. For all experiments, mice were used at 3–4 or >18 months of age. Both males and females were used, except for the neurogenesis experiments, where only males were tested. Mice were kept on a 12-hr light/dark cycle with access to food and water *ad libitum*. Lithium diet was prepared by adding lithium carbonate (2.4 g/kg) to animal food and purchased from Bio-Serve, Inc. Animals were fed lithium diet for one month and closely monitored for any health issues. The Institutional Animal Use and Care Committee of the Cleveland Clinic approved all experiments. Animals were euthanized following CCF IACUC approved protocol by CO2 asphyxiation followed by cervical dislocation. Animals were monitored daily for CCF veterinary staff. We had a place in protocol for early/humane endpoints used and approved by AALAC, CCF IACUC and Veterinary experts. All animals were monitored daily for any signs of distress. Appropriate methods were used to minimize/alleviate animal suffering. All experiments were performed by examiners blinded to the genotypes or treatments of the mice.

### Kainic acid injections

Kainic acid (Sigma) was prepared as a 1.0 mg/ml solution in normal saline (0.9 M NaCl) and injected intraperitoneally (i.p.) at a dose of 20 mg/kg body weight [17]. Mice given sub-lethal doses were sacrificed after 3 days and transcardially perfused with ice cold Phosphate-buffered saline (PBS) followed by 4% paraformaldehyde (PFA) and processed as described below.

### Assessment of excitotoxicity

Kainic acid (KA)-injected mice were placed in a cage and observed for 60 min after KA administration. Seizure severity was scored according to a modified Racine scale as described previously [17]. Briefly, scores were: 0 = normal behavior; 1 = immobility; 2 = generalized spasm, tremble, or twitch; 3 = tail extension; 4 = forelimb clonus; 5 = generalized clonic activity; 6 = bouncing or running seizures; 7 = full tonic extension; 8 = death. An investigator blinded to genotype quantified the time course and severity of seizures.

### Immunohistochemistry

Hemi-brains were fixed in PBS containing 4% PFA overnight, sunk in 30% sucrose and embedded in OCT. Sagittal sections were cut (30 μm) and every 12^th^ section was used for immunohistochemistry. Sections were washed in PBS, treated with 3% H_2_O_2_ in PBS and incubated in blocking solution (5% normal goat serum in PBS containing 0.01%Triton-X) for 1 h at room temperature. Sections were incubated with primary antibodies against NPY (1:1000), AT-8 (1:500), At-180 (1:500) or DCX (Millipore, 1:1000) overnight at 4°C followed by respective secondary antibodies (1:250) and developed using the ABC kit (Vector Labs). Microscopy was performed using a Leica DMR microscope equipped with a CCD camera for bright field imaging.

### Western Blots

Hippocampal and cortical tissue was isolated from mice at 3–4 months of age and homogenized in tissue lysis buffer. After blocking, membranes were incubated in primary antibodies overnight at 4°C. Primary antibodies used were anti total-NMDAR antibody (Millipore, 1:2000), anti p-NMDAR (Pierce, 1:1000), AT-8 (Pierce, 1:1000), AT-180 (Pierce, 1:1000), Tau5 (Pierce, 1:1000) and GAPDH (Chemicon, 1:40000). ImageJ was used for densitometric analysis and bands were normalized to GAPDH loading controls for each sample.

### Cell Counting

Cell counting from hippocampus was performed as described previously [16]. Confocal images of hippocampal CA3 were taken with a 40x objective using a confocal microscope (Leica SP5). Boxes of the same area were drawn over each region and the selected region was counted for NeuN-positive cells using the cell counter plug-in from ImageJ. Every 12^th^ section was stained for NeuN and counted and quantified as the number of NeuN-positive cells/area (mm^2^).

### Y maze

The Y-maze was constructed of black plastic walls 10 cm high with 3 identical arms (21 cm length x 8.5 cm width x 40 cm height) positioned at 120 degrees from each other. The mouse was placed in one of the compartments and allowed to move freely for 5 min. An arm entry was manually recorded when all four paws entered the compartment. Spontaneous alternations (SAs) are the subsequent entry into a novel arm over the course of 3 entries and the % SAs were calculated as [(number of SAs/total arm entries)-2] x 100. Total arm entries were also assessed as a measure of hyperactivity. After testing each mouse, the maze was thoroughly cleaned to standardize odors.

### 5-Bromo-2-deoxyuridine (BrdU) injection and stereological counts

BrdU injections and counts were performed as described previously [23, 24]. To avoid the effects of gender and gonadotropic hormones on adult neurogenesis, only male mice were used. To determine cell proliferation, mice were injected once daily with BrdU (100 mg/kg, i.p.) for three consecutive days. On the day following the last BrdU injection, animals were anesthetized and transcardially perfused with ice-cold PBS followed by 4% PFA. Brain were removed and fixed in 4% PFA overnight at 4°C, cryo-protected in 30% sucrose, embedded using OCT compound (Sakura Finetek, Torrance, CA, USA) and stored at -80°C. The total number of BrdU+ cells in the subgranular zone of the dentate gyrus was quantified using unbiased stereological methods. BrdU+ cells were counted from every sixth section using a 40X objective throughout the entire rostro-caudal extent of the dentate gyrus (bregma −1.0 mm to −2.80 mm). Cells were counted from both halves of the brain within the granular cell layer (GCL) and adjacent SGZ up to a two-cell body-wide zone along the border between the GCL and the hilus. The experimenter counting the cells was blinded to the genotype and experimental modification of the mice. The total number of cells was obtained by multiplying the number of BrdU+ cells with inter-section interval and adding them together for the entire hippocampus.

### Statistical analysis

Statistics were performed using Graphpad Instat software (version 4). One-way ANOVA was employed and significance was set at *p*<0.05. Bar graphs show mean ± SEM.

## Results

### Lack of endogenous tau prevents defective neurogenesis in AICD-Tg mice

**Fig 1:**
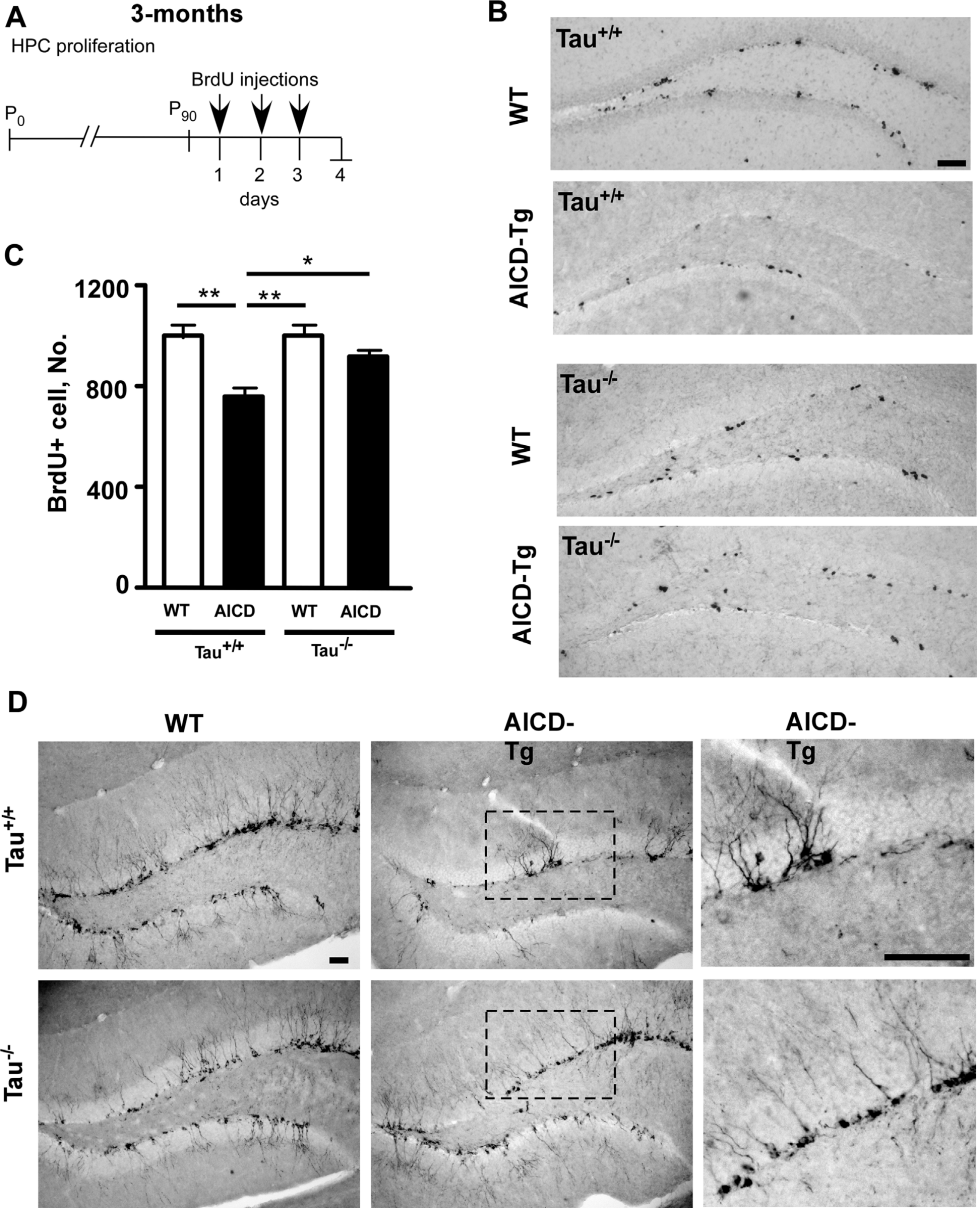
Lack of tau prevents impaired adult hippocampal progenitor cell proliferation in AICD-Tg mice. A. Experimental design to measure hippocampal neurogenesis. After the last BrdU injection (100 mg/kg), mice were sacrificed the next day (proliferation). B. Representative images of BrdU immunostaining in the subgranular zone (SGZ) of the dentate gyrus performed on 3-month-old wild-type and AICD-Tg animals with or without endogenous mouse tau (Tau+/+ or Tau-/-, respectively). C. Quantitative analysis of the total number of BrdU+ cells throughout the entire rostro-caudal extent of the hippocampus revealed a statistically significant decrease in the number of BrdU+ cells in the SGZ of AICD-Tg mice compared to wild-type mice. Lack of tau (Tau-/-) protects against cell proliferation deficits in AICD-Tg. D. Coronal sections from brains of 3-month-old wild-type and AICD-Tg mice were immunostained for the immature neuronal marker doublecortin (DCX). Compared to wild-type (left) there is a significant decrease in the number of DCX+ cells in the SGZ of AICD-Tg mice (right). Tau-/- protects against impaired neurogenesis in AICD-Tg mice (bottom). Tau-/- mice alone had DCX staining similar to WT mice in the hippocampus. Boxed regions are enlarged to show differences in the number of DCX+ immature neurons in the dentate gyrus. n = 5 for both wild-type and AICD-Tg mice in Tau+/+ background and 7 and 6 respectively in Tau-/- background. * p<0.05, ** p<0.01 One-way ANOVA. Scale bar = 100 μm.

In the adult mammalian brain new neurons are born in the hippocampus and in the sub-ventricular zone lining the lateral ventricles [25]. Hippocampal neurogenesis has been implicated in learning and memory and is impaired in AD [26]. Hippocampal neurogenesis is also impaired in several Aβ-based mouse models of AD and in AICD-Tg mice [27]. To study the role of tau in AICD-driven impaired neurogenesis, we crossed AICD-Tg mice with Tau^-/-^ mice [22] and F1 mice (AICD/Tau^+/-^) were interbred to generate AICD/Tau^-/-^. Three-month-old mice of various genotypes were injected with three consecutive daily injections of 3-Bromo-5’-deoxy uridine (BrdU) (Fig 1A) and BrdU immunohistochemistry was performed to measure hippocampal progenitor cell (HPC) proliferation one day following the last injection. As reported previously [23, 24], 3-month-old AICD-Tg mice exhibited reduced BrdU+ cells compared to wild-type animals (Fig 1B and 1C). Knocking out endogenous mouse tau (Tau^-/-^) significantly reduced the deficit in HPC proliferation in AICD-Tg mice (Fig 1B and 1C), such that HPC proliferation in AICD-Tg/Tau^-/-^ mice was comparable to WT mice.

We also previously showed that AICD-Tg mice exhibit reduced numbers of doublecortin (DCX)-positive cells in the hippocampus as early as 3 months of age [23, 24]. DCX is expressed by immature neurons but not by glia. AICD-Tg mice showed a reduction in the number of DCX-positive cells in the adult hippocampus (Fig 1D). However, lack of tau prevented the loss of DCX-positive immature neurons in the hippocampus of AICD transgenic animals (Fig 1D, right panels). Together, these results indicate that lack of tau can rescue the deficit in adult neurogenesis observed in AICD-Tg mice.

### Lack of tau protects against kainic acid-induced seizure susceptibility, excitotoxicity and neurodegeneration

**Fig 2:**
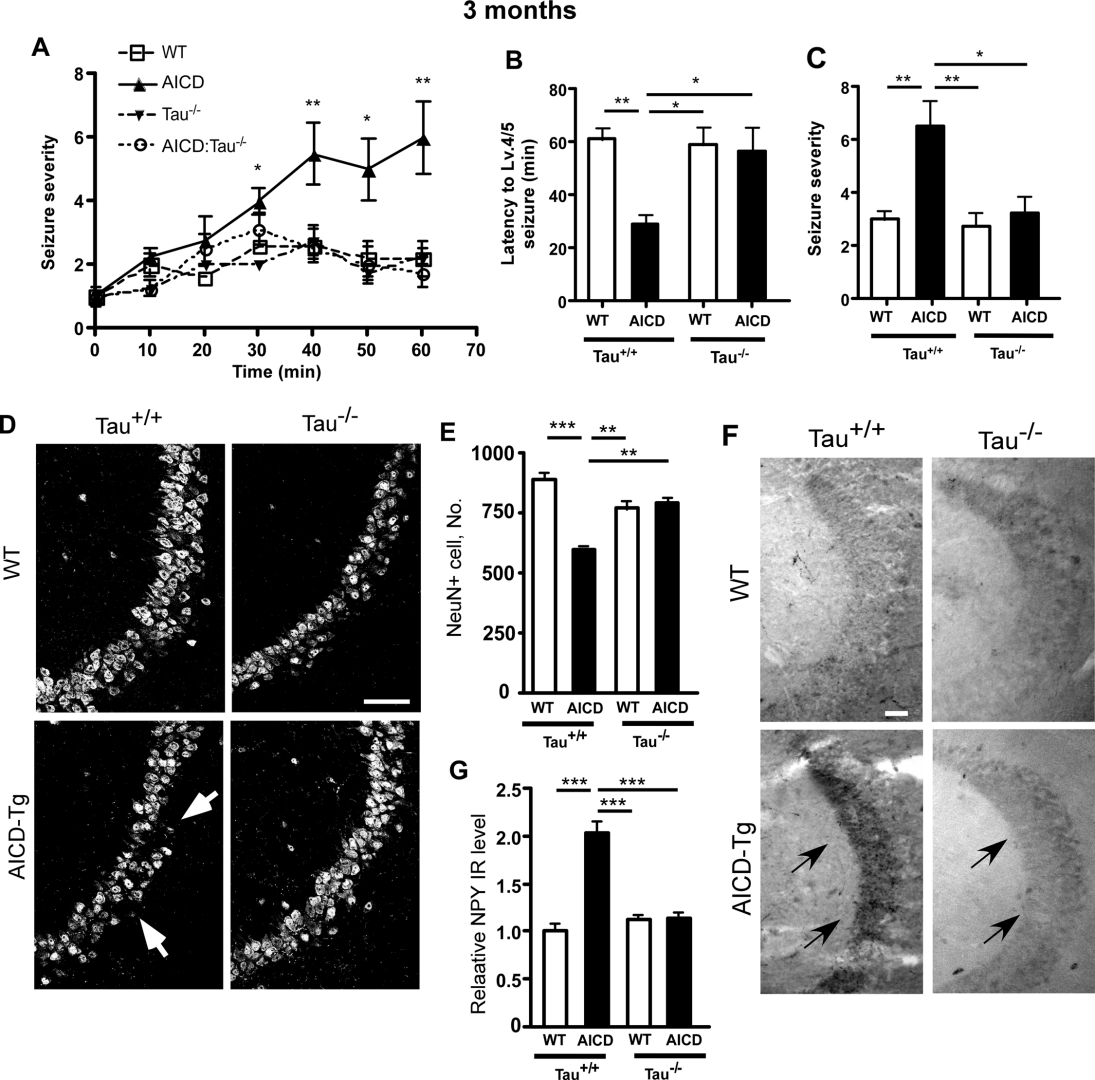
Tau knockout prevents sensitivity to Kainic acid–mediated stress in AICD-Tg mice. A. AICD-Tg mice exhibit increased sensitivity towards Kainic Acid (KA)-mediated seizures at 3–4 months of age. Animals were injected with a sub-threshold dose of KA (20 mg/kg) and observed for 60 minutes for seizure–like hyperactivity. Seizures were scored according to a modified Racine scale. AICD-Tg mice began to show significant signs of seizures at 30 min. However, lack of tau (Tau-/-) significantly protected AICD-Tg mice against KA-mediated seizure-like hyperactivity. Tau-/- mice behaved similar to wild-type (WT) littermates. B. The time taken to reach level 4–5 seizures was scored as the latency to reach convulsive seizures. Lack of tau (Tau-/-) had a protective effect on AICD transgenic animals. C. Mean seizure severity scores showed a significant rescue of seizure severity by Tau-/- in AICD animals. n = 4–5 mice per group (A-C). D. Sagittal sections from 4-month-old WT and AICD-Tg mice were stained with NeuN antibody. Arrows show the loss of neurons in the CA3 region of AICD-Tg mice. Deletion of Tau protected AICD-Tg mice from KA-mediated neuronal loss. E. Quantification of NeuN-positive cells shows that Tau-/- makes AICD-Tg mice resistant to KA-mediated neurodegeneration. F. AICD-Tg mice show an increase in neuropeptide-Y (NPY) expression in mossy fiber terminals (arrows) following KA-mediated seizures. Tau deletion decreased NPY expression in mossy fiber terminals following KA injection in AICD-Tg animals. G. Quantification of NPY immunoreactivity normalized to WT animals. *p<0.05, **p<0.01, ***p<0.001, one way ANOVA (all data expressed as Mean ± SEM). n = 5 for all groups (D-G). Scale bar = 50 μm (D) and100 μm (F).

Seizure-like hyperactivity has been detected in a subset of the AD population. Seizure-like hyperactivity has also been detected in some Aβ-based mouse models of AD (reviewed in [28]), but reduction of tau has been shown to protect against these pathologies in mouse models of AD [11, 21, 28–31]. AICD transgenic mice also exhibit increased vulnerability towards the seizure-inducing drug kainic acid (KA) [16, 18]. To test the involvement of tau in hyperactivity and excitotoxicity, mice of various genotypes were injected i.p. with KA at 20 mg/kg and observed for seizure progression. Forty minutes after KA injection, AICD-Tg mice showed a significant increase in seizure score compared to WT littermates (Fig 2A and 2C and S4 Fig), eventually exhibiting signs of tonic-clonic seizures. By contrast, AICD;Tau^-/-^ mice behaved similarly to Tau^-/-^ mice or WT littermates and had significantly lower seizure severity scores than AICD-Tg mice alone (Fig 2A and 2C). We also measured the latency to reach level 4/5 seizures and found that AICD-Tg mice took significantly less time in reaching level 4/5 seizures than AICD;Tau^-/-^, Tau^-/-^ or WT littermates (Fig 2B). These data suggest that lack of tau exerts protective effects on AICD-mediated seizures or vulnerability to KA-mediated seizures.

A previous *in vitro* study reported that AICD renders cultured primary neurons vulnerable to excitotoxic stress [32] whereas we previously showed that KA induces significant neurodegeneration *in vivo* in AICD-Tg mice [16]. Since genetic ablation of tau protected against seizure-like hyperactivity in AICD transgenic mice, we next tested whether lack of tau could protect against KA-induced excitotoxic insults. 3-4-month-old mice were administered a sub-lethal dose of KA (20 mg/kg), sacrificed 3 days later and brain sections were stained with anti-NeuN antibodies for neuronal nuclei in the CA3 region as described previously [16]. We observed a significant reduction in the number of CA3 neurons in AICD-Tg mice compared to age-matched KA-treated WT littermates (Fig 2D and 2E). However, AICD-Tg mice lacking tau were resistant to neuronal loss resulting from KA-mediated excitotoxic stress and had similar numbers of neurons present as compared to Tau^-/-^ littermates or WT animals (Fig 2D and 2E).

We next determined the levels of Neuropeptide Y (NPY), which is upregulated in response to seizures in mossy fiber terminals [33] and is also found to be upregulated in mouse models of AD with seizures, such as hAPP-J20 and PS1/APP [34]. We previously showed that AICD-Tg mice have enhanced NPY expression in mossy fiber terminals compared to WT controls after KA-induced seizures [18]. Here we tested whether heightened NPY expression after KA injection was reduced in AICD-Tg mice on the Tau^-/-^ background. Consistent with our previous findings, AICD-Tg mice had increased expression of NPY in mossy fiber terminals in response to a sub-lethal dose of KA compared to WT littermates (Fig 2F and 2G). However, AICD;Tau^-/-^ mice showed a moderate expression of NPY that was much less than that observed in AICD-Tg mice (Fig 2F and 2G). Essentially, the NPY expression in AICD-Tg;Tau^-/-^ mice was similar to Tau^-/-^ or WT controls after KA administration (Fig 2G). These results collectively indicate that lack of tau confers protection against overt seizure-like phenotypes, hippocampal remodeling and downstream pathological changes in AICD-Tg mice.

### Lack of tau normalizes hyperphosphorylation of NMDA receptors in AICD-Tg mice

**Fig 3:**
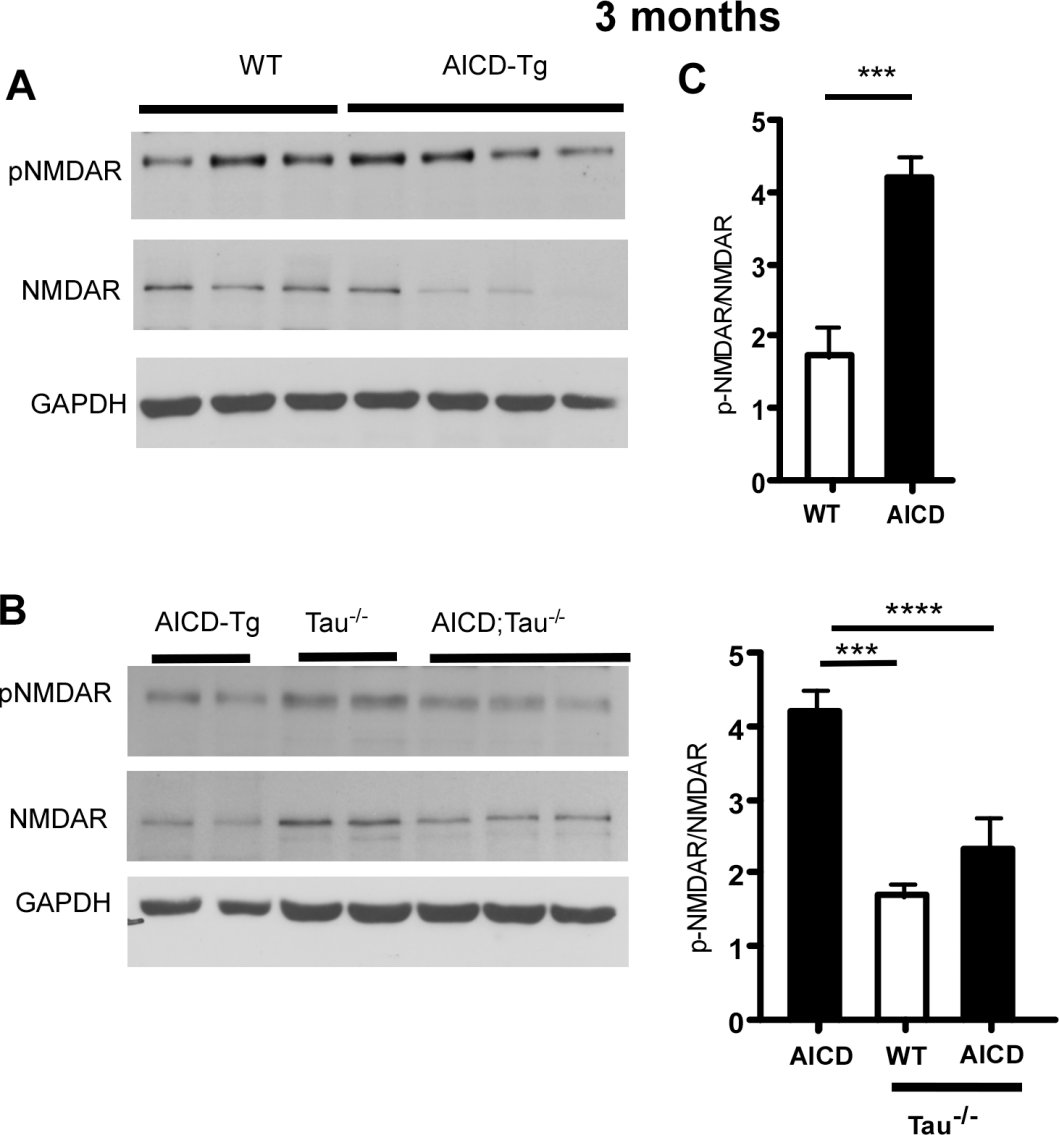
Lack of tau normalizes increased p-NMDAR levels in AICD animals. A. Representative immunoblots showing that AICD-Tg mice at 3–4 months of age have increased pY1472-NR2B (pNMDAR) levels to total NMDAR levels compared to age-matched non-transgenic animals. B. Lack of tau normalizes the increased pNMDAR levels in AICD-Tg animals. C. Quantification of pNMDAR/NMDAR levels in wild-type and AICD animals in the presence (Tau +/+) or absence (Tau-/-) of tau. n = 5–6 for each genotype. *p<0.05, **p<0.01, ***p<0.001 one way ANOVA (Mean ± SEM).

Tau has been shown to mediate the harmful effects of Aβ, although the precise mechanism is not clearly understood [7, 28]. Recently, it was suggested that the toxic effects of Aβ are mediated by a dendritic function of tau involving increased levels of phosphorylated NMDA receptor (NMDAR) [12]. To test whether the excitotoxicity observed in AICD-Tg animals is dependent on NMDARs, we examined the levels of NMDAR and that of NMDAR phosphorylated at tyrosine 1472 of the NR2B subunit (pNMDAR) that controls the activity of NMDAR [35, 36]. At 3 months of age AICD-Tg mice showed significantly decreased levels of NMDAR and an increased ratio of phosphorylated NMDAR to total NMDAR (Fig 3A and 3C). Interestingly deletion of tau prevented the hyperphosphorylation found in AICD-Tg mice (AICD-Tg;Tau^-/-^), such that the ratio of p-NMDAR/total NMDAR was similar to that observed in tau^-/-^ mice (Fig 3B and 3C). We also examined AMPA receptors (GRIA1 and GRIA2) in WT and AICD-Tg mice and detected no significant changes in the levels of either GRIA1 or GRIA2 (S1 Fig). These results show that there is increased phosphorylation of NMDAR in AICD-Tg animals and that loss of tau results in a normal p-NMDAR/NMDAR ratio in AICD-Tg animals. Taken together, these results suggest that tau acts as a mediator of many downstream pathologies of AICD *in vivo*, including NMDAR levels.

### Reduced spontaneous alternation in Y-maze test of working memory in aged AICD-Tg mice is rescued by lack of tau

**Fig 4:**
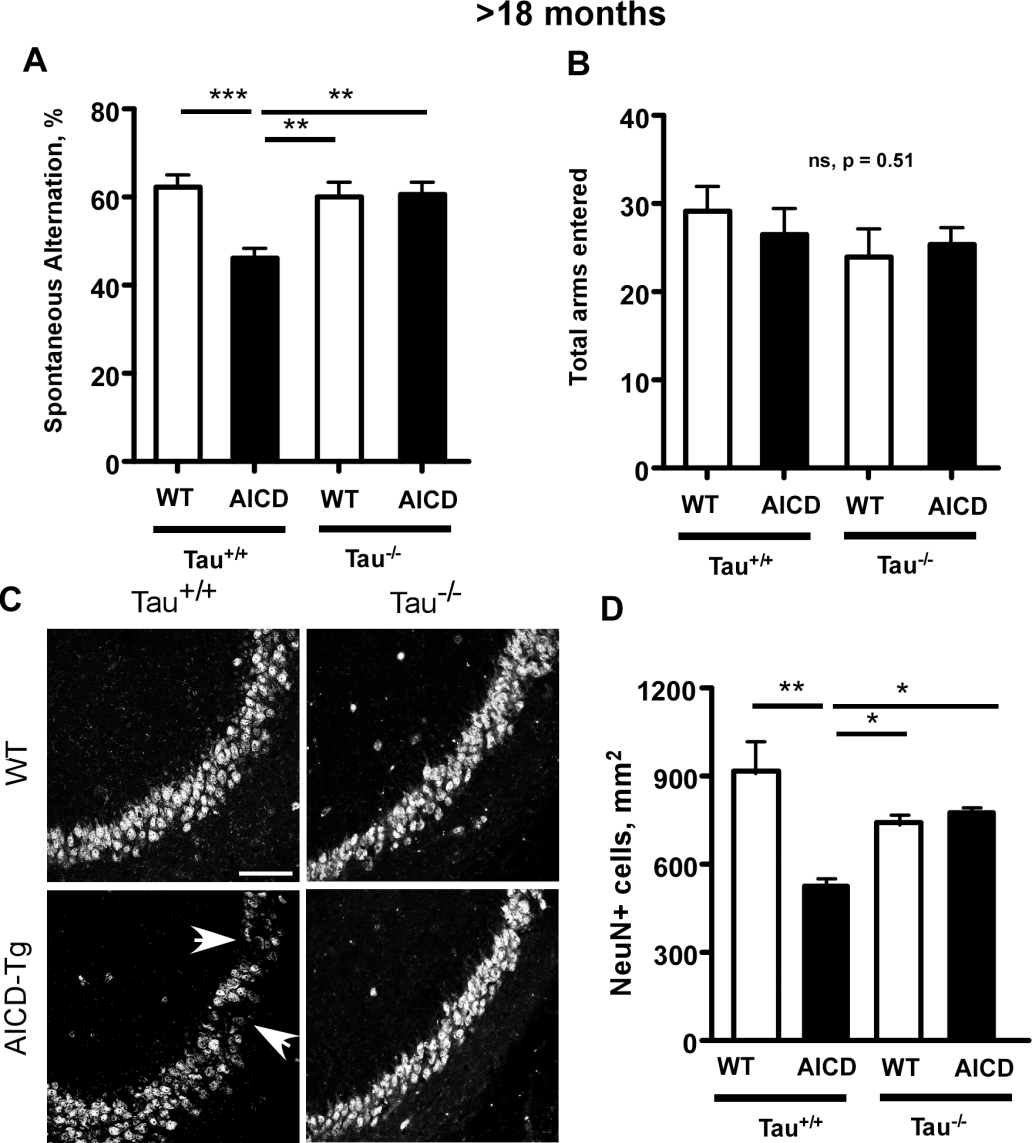
Deficiency of tau protects against age-dependent neurodegeneration and memory loss measured by deficits in spontaneous alternations in AICD-Tg mice. A. AICD-Tg mice exhibit decreased working memory as measured by deficits in spontaneous alternations at older ages. At >18 months of age, AICD-Tg mice showed decreased spontaneous alternations (working memory) compared to age-matched wild-type animals. Lack of tau (Tau-/-) was able to protect AICD-Tg mice from such deficits in working memory. B. Normal exploratory behavior for all the genotypes measured in the Y-maze. n = 10–12 mice per group for behavioral experiments (A and B). C. AICD-Tg mice at more than 18 months of age showed neuronal loss as assessed by NeuN staining in the CA3 region. Arrows point to focal loss of neurons. Genetic ablation of tau (Tau-/-) protected against neurodegeneration in AICD-Tg mice. D. Quantification of NeuN-positive nuclei. n = 4 for all groups. *p<0.05, **p<0.01 one way ANOVA (Mean ± SEM). Scale bar = 50 μm. WT = wild-type.

We previously showed that AICD-Tg mice begin to show memory deficits as measured by deficits in spontaneous alternations starting at 7–8 months of age [16], which persists at older ages. To determine whether lack of tau also protects AICD-Tg mice against memory deficits, we subjected older animals to the Y-maze test. At > 18 months of age, AICD-Tg mice showed a significant decrease in the percentage of spontaneous alternations in the Y-maze test compared to WT mice (Fig 4A). We ascertained that these changes were not due to altered motor function because the number of total arms entered (a measure of normal exploratory behavior) was similar for both AICD-Tg and WT mice (Fig 4B). However, AICD-Tg;Tau^-/-^ mice did not show any changes in spontaneous alternations compared to WT or Tau^-/-^ littermates and had significantly better working memory compared to age-matched AICD-Tg mice (Fig 4A). These data suggest that deletion of tau protected against short-term memory loss measured by spontaneous alternations in Y-maze normally observed in aged AICD-Tg mice at greater than 18 months of age.

### Lack of Tau prevents age-dependent neurodegeneration in AICD-Tg mice

Multiple lines of evidence indicate that AICD-Tg mice are prone to stress associated with aging or excitotoxicity [16, 18]. KA administration in young AICD-Tg mice recapitulated excitotoxic and neurodegenerative changes observed in naive, aged AICD-Tg mice, suggesting that elevated levels of AICD rendered neurons vulnerable to aging-associated stress [16]. Since deletion of tau protected AICD-Tg mice from KA-induced neurodegeneration, we next examined whether lack of tau could protect AICD-Tg mice from age-related neurodegeneration. At >18 months of age, there was a significant loss of neurons in the CA3 region of AICD-Tg mice compared to age-matched WT mice (Fig 4C (arrows) and 4D). Deletion of endogenous mouse tau protected AICD-Tg mice from such neuronal loss (Fig 4C and 4D). Aged AICD;Tau^-/-^ mice had more neurons present in the CA3 region compared to age-matched AICD-Tg mice (Fig 4D). Together, these findings show that lack of tau protects AICD-Tg mice from the deleterious effects of accumulated AICD and suggest that tau mediates the deleterious effects of AICD, as reported in other Aβ-based models of AD [28].

### Increased levels of human tau enhance somato-dendritic tau accumulation and NR2B phosphorylation in AICD-Tg animals

**Fig 5:**
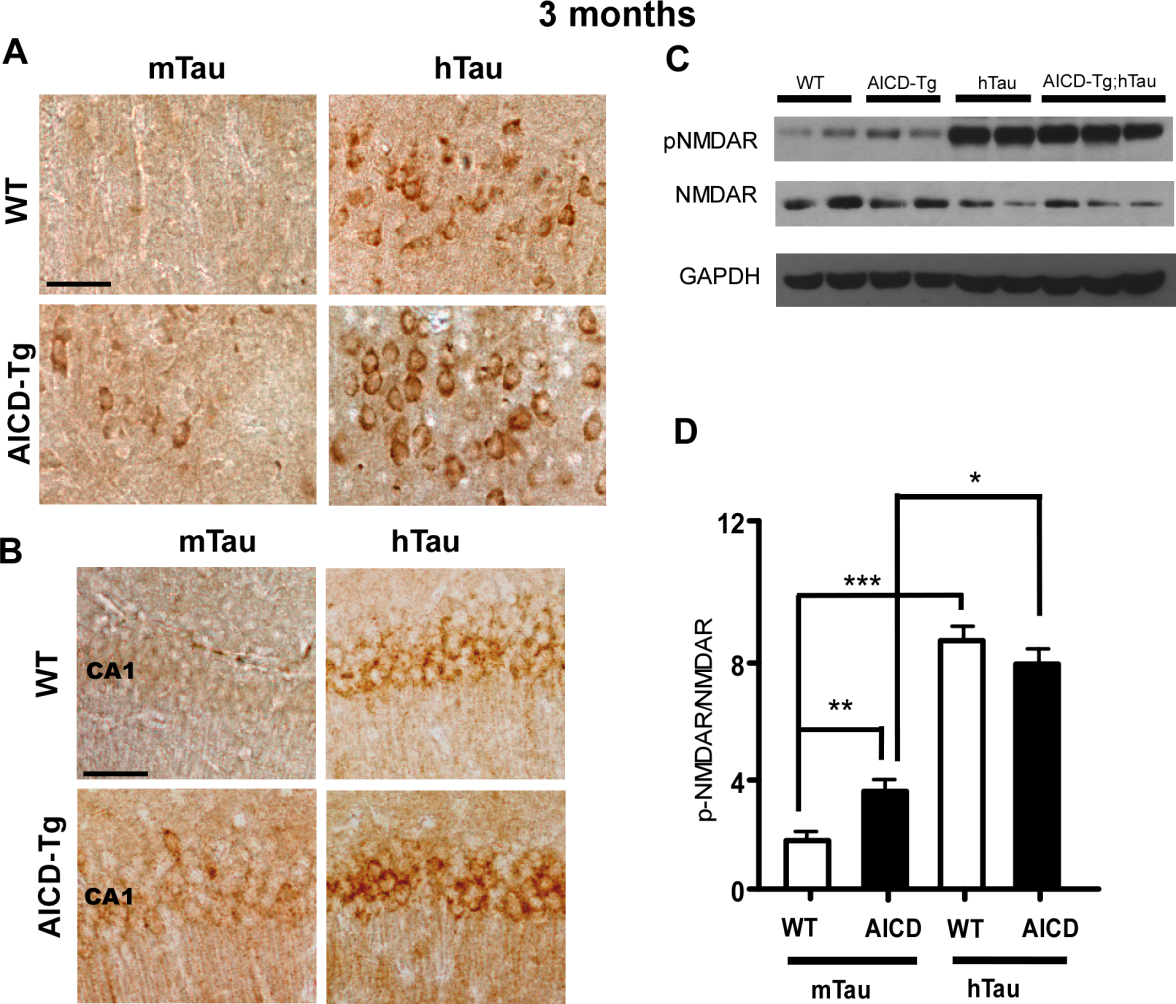
Increased somato-dendritic tau accumulation and phosphorylation of NMDAR after hTau overexpression. A-B Phospho-tau staining with AT8 (A) and AT180 antibody (B) revealed increased somato-dendritic accumulation of phosoho-tau in the cerebral cortex (A) and hippocampal CA1 layer (B) of hTau animals compared to wild-type (WT) and AICD-Tg transgenic mice at 3–4 mo of age. Scale bar = 100 μm C. Overexpression of human tau (hTau) increased pY1472-NMDAR levels in both WT and AICD animals. D. Quantification of pY1472-NMDAR/NMDAR levels in wild-type and AICD animals in the presence of mouse (mTau) or human tau (hTau). n = 5–6 for each genotype. *p<0.05, **p<0.01 one way ANOVA (Mean ± SEM). Sale bar in A,B = 50 μm.

Data presented above indicate that lack of tau ameliorates the AD-like pathologies in AICD-Tg mice. This raised the possibility that increasing the levels of tau could exacerbate the pathological features in AICD-Tg animals. To address this possibility, we crossed AICD-Tg mice with hTau mice [22] that overexpress full-length wild-type human tau. Thus, this AICD;htau double transgenic mouse model overexpresses human tau in the presence of endogenous mouse tau. Since a number of tau-mediated pathologies in AD depend on somato-dendritic accumulation of tau [28], we first determined the levels of somato-dendritic tau accumulation in hTau overexpressing mice. Compared to WT and AICD animals, hTau and AICD;hTau animals displayed higher levels of somato-dendritic accumulation of phosphorylated-tau in the cerebral cortex (Fig 5A) and in the hippocampus (Fig 5B). Western blot analysis revealed that hTau animals had higher levels of phosphorylated tau (S2 Fig). In addition, overexpression of human tau increased p-NMDAR/total NMDAR levels in AICD transgenic mice (Fig 5C and 5D). Interestingly, overexpression of human tau alone was sufficient to increase p-NMDAR levels in WT mice (Fig 5C and 5D). These results indicate that overexpression of human tau, which correlates with increased somato-dendritic accumulation of phosphorylated tau, results in increased phosphorylation of NMDAR.

### Human tau overexpression exacerbates impaired neurogenesis in AICD-Tg mice

**Fig 6:**
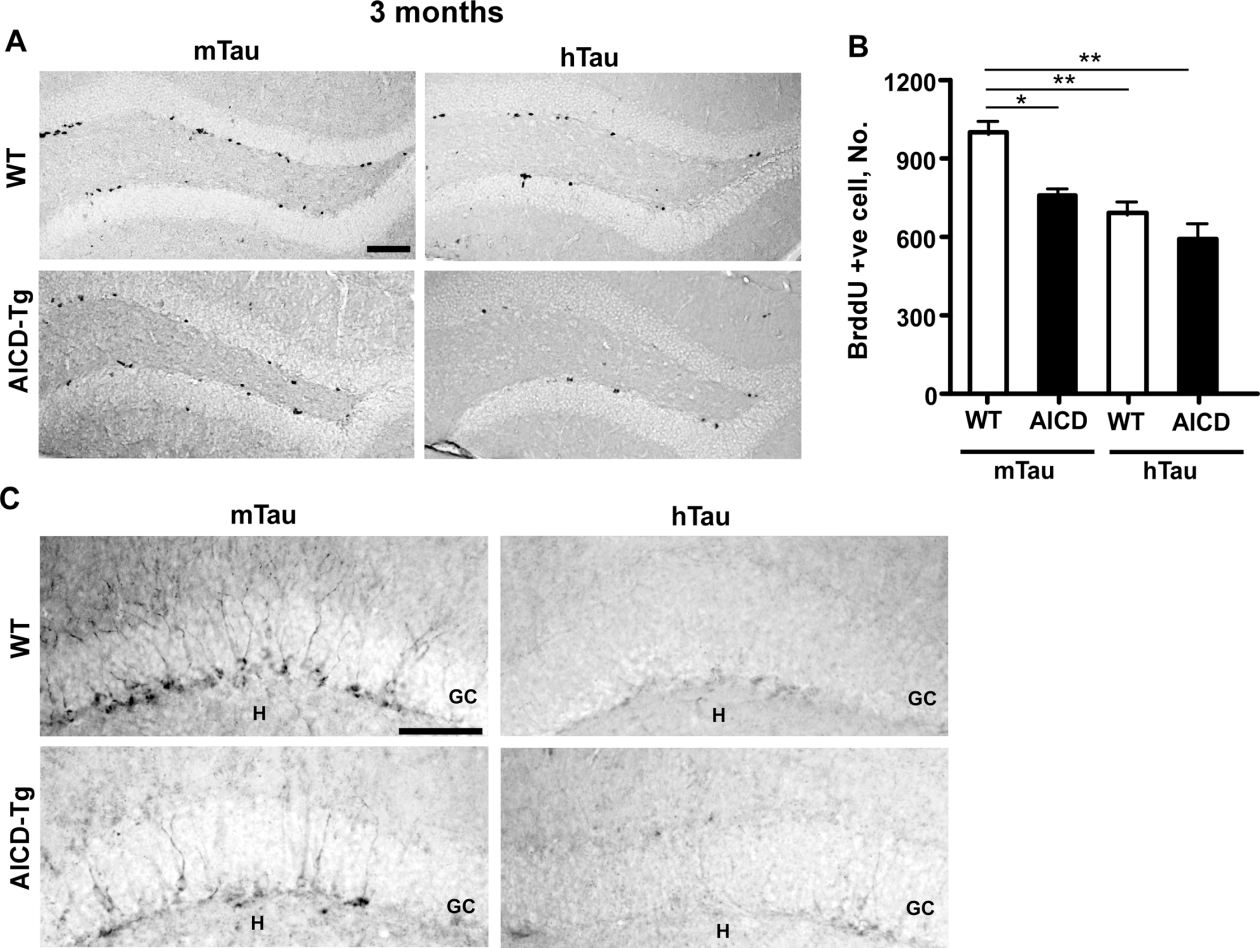
Human tau overexpression impaired adult hippocampal progenitor cell (HPC) proliferation. A. Representative images of BrdU immunostaining in the subgranular zone (SGZ) of the dentate gyrus performed on 3-month-old wild-type and AICD-Tg animals with endogenous mouse tau (mTau) or overexpressed human tau (hTau). B. Quantitative analysis of the total number of BrdU+ cells throughout the entire rostro-caudal extent of the hippocampus in 3-month-old animals revealed a statistically significant decrease in the number of BrdU+ cells in the SGZ of AICD-Tg mice compared to wild-type mice. However, overexpression of human tau (hTau) alone also decreased HPC proliferation in wild-type animals and had a tendency to decrease HPC proliferation in AICD animals. n = 4–7 for each group. C. Representative images of doublecortin (DCX) immunostaining on 3-month-old wild-type and AICD-Tg animals with endogenous mouse tau (mTau) or overexpressed human tau (hTau) revealed a decrease in the number of immature neurons in both WT and AICD animals that overexpress h-Tau. GC: Granule cell layer; H: Hilus. *p<0.05, **p<0.01 one way ANOVA (Mean ± SEM). Scale bar = 100 μm.

We next examined the status of hippocampal adult neurogenesis in AICD;hTau mice. Expression of human tau alone was sufficient to significantly reduce hippocampal progenitor cell proliferation in WT animals by 24% (Fig 6A and 6B) at 3–4 months of age. Moreover, human tau expression had a tendency to decrease HPC proliferation by 20% in AICD transgenic mice of the same age (Fig 6A and 6B). Doublecortin staining for immature neurons revealed reduced immunostaining for hTau and AICD;hTau mice compared to WT and AICD-Tg mice at 3 months of age (Fig 6C). This suggests that increased levels of tau result in the decreased adult neurogenesis seen in AD.

### Human tau overexpression causes behavioral deficits and neurodegeneration

**Fig 7:**
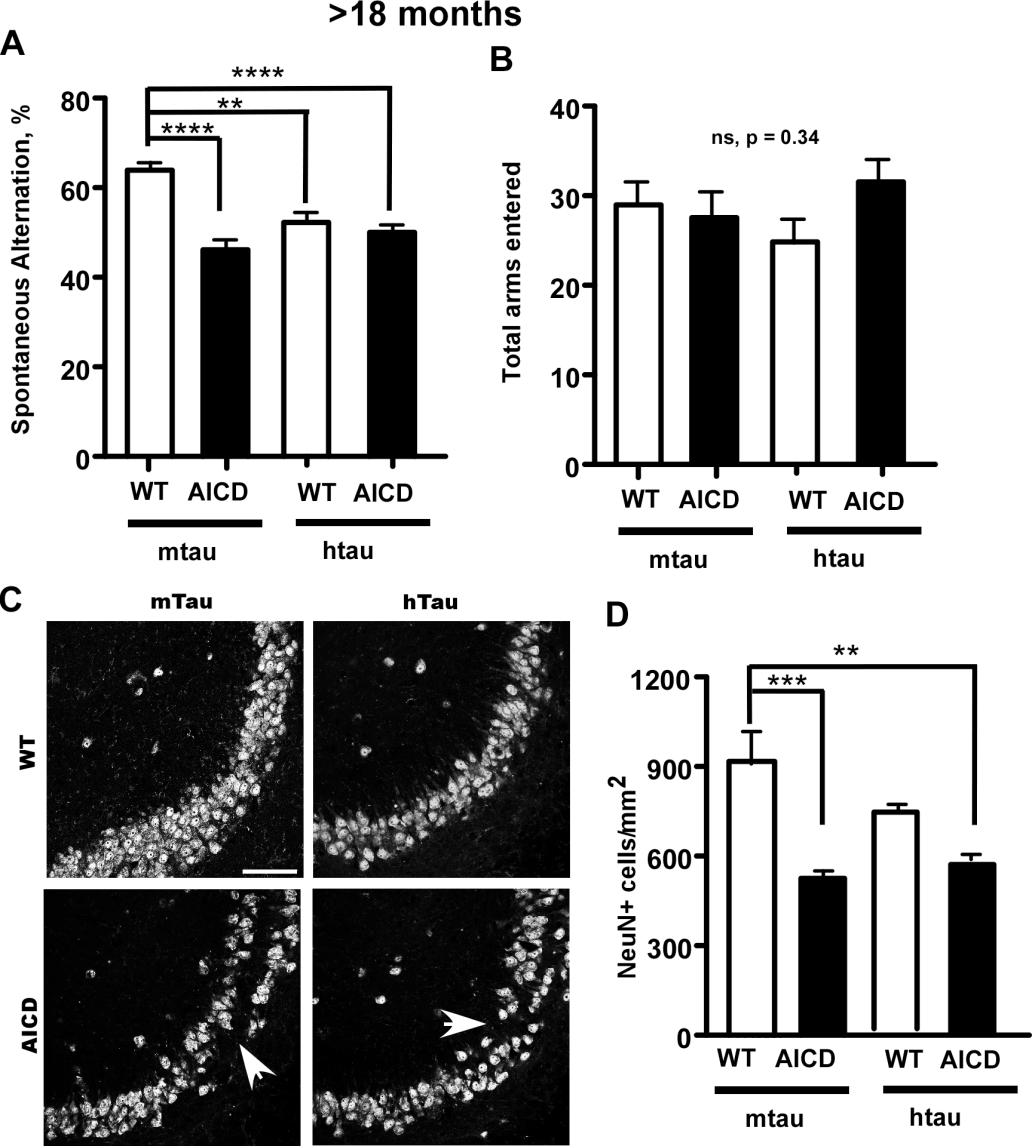
Tau overload causes behavioral deficits and maintains neurodegeneration at older ages. A. hTau animals also showed decreased working memory as measured by deficits in spontaneous alternations compared to age-matched wild-type animals. AICD-Tg mice exhibit decreased working memory at older ages compared to age-matched wild-type animals. B. Evidence of normal exploratory behavior was demonstrated for all the genotypes measured in the Y-maze. n = 10–12 mice per group for behavioral experiments (A and B). C. Old AICD-Tg mice carrying an overload of human tau showed neuronal loss similar to age-matched AICD-Tg mice without human tau as assessed by NeuN staining in the CA3 region. Arrows point to focal loss of neurons. Though not statistically significant, there was a trend toward hTau mice having less neuronal cell bodies in the CA3 compared to age-matched wild-type animals. D. Quantification of NeuN-positive nuclei. n = 5 for all. *p<0.05, **p<0.01, ***p<0.001 one way ANOVA (Mean ± SEM). Scale bar = 50 μm

Since overexpression of hTau exacerbated the deficits in adult neurogenesis, we next tested the effects of tau overexpression on spontaneous alternation and neurodegeneration at older ages (>18 months). In the Y-maze test for working memory measured by deficits in spontaneous alternations, aged hTau animals showed impaired short-term memory compared to non-transgenic animals (Fig 7A). We already observed that AICD also impairs short term-memory at older ages (Figs 2A and 7A). Human tau overexpression did not exacerbate the impaired memory in AICD-Tg mice, thereby showing no additive effects (Fig 7A). We found no difference in exploratory behavior amongst the four groups (Fig 7B), suggesting there were no gross motor impairments.

We next examined brain sections with anti-NeuN antibodies and found that overexpression of human tau alone tended to reduce the number of CA3 neurons at older ages in AICD transgenic mice (Fig 7C and 7D). Human tau did not have an additive effect on neurodegeneration in the CA3 region seen in AICD transgenic mice (AICD;hTau Fig 7C and 7D). These results suggest that tau overload has deleterious effects on some behaviors and neurodegeneration which were not additive to those caused by AICD.

### Inhibiting tau hyperphosphorylation with lithium treatment ameliorates impaired neurogenesis in AICD-Tg mice

We previously showed that GSK-3β activity is stimulated in AICD-Tg mice and that inhibiting GSK-3β activity with lithium can protect against behavioral deficits in AICD animals [16]. GSK-3β hyperphosphorylates tau, which then detaches from microtubules and accumulates in the somato-dendritic compartment. Since somato-dendritic accumulation of tau leads to increased phosphorylation of NMDAR and subsequent toxicity [12, 37, 38], we tested whether blocking GSK-3β activation could rescue AICD-mediated pathologies.

**Fig 8:**
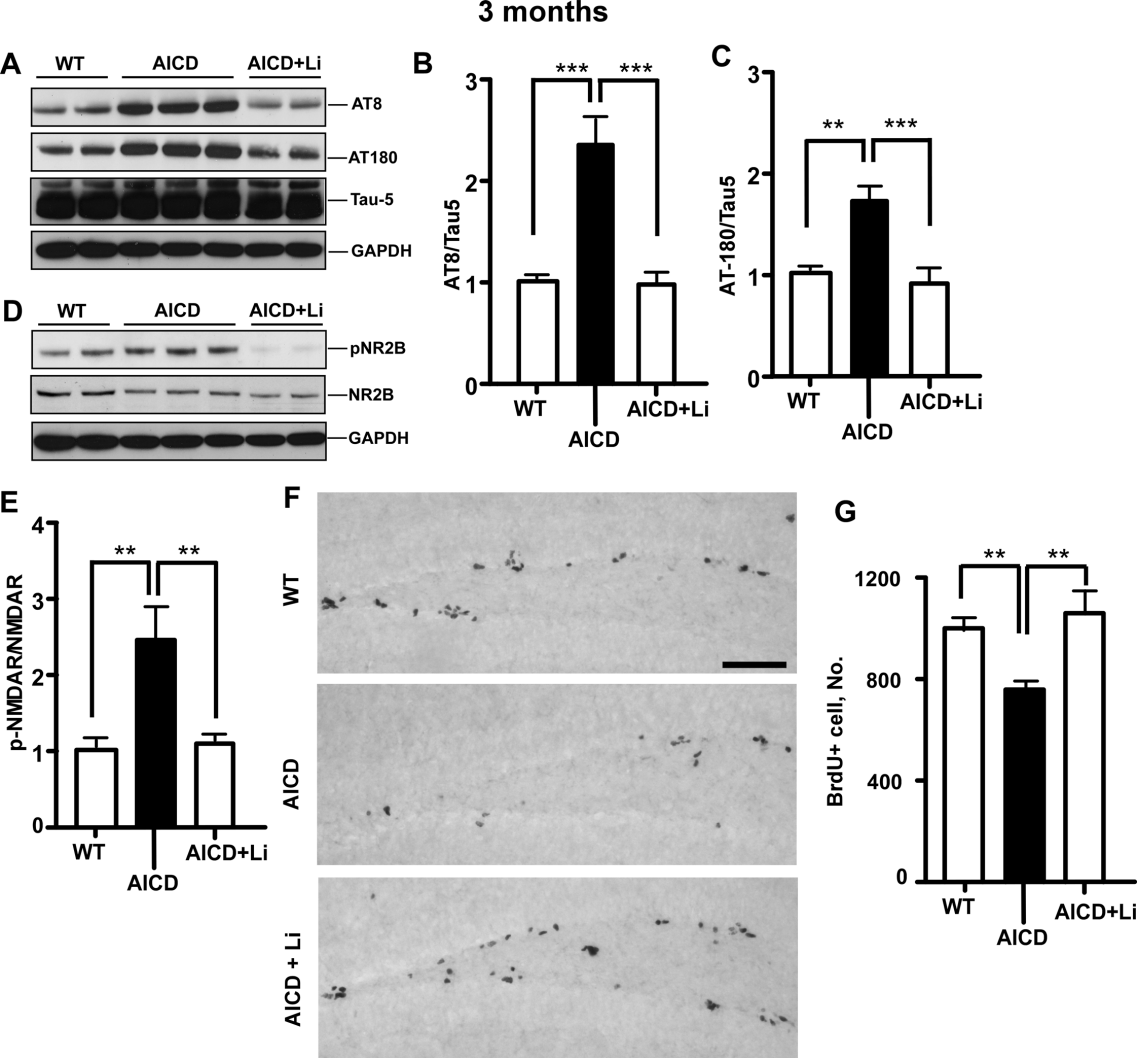
Inhibiting GSK-3β protects against tau pathology and rescues impaired neurogenesis in AICD-Tg mice. A. Hippocampal brain lysates from 3-month-old wild-type (WT) mice, AICD-Tg mice and AICD-Tg mice fed lithium chow (AICD+Li) were western blotted using AT8, AT180 or Tau-5 to detect phospho- tau or total tau. AICD animals showed increased phospho-tau compared to WT mice, while total tau levels were not significantly different. Inhibiting the tau kinase GSK-3β with lithium treatment for a month decreased the amount of phosphorylated tau in the hippocampus of AICD-Tg mice. B-C. Quantification of western blots for AT8 and AT180 respectively, showed a significant reduction in the amount of phospho-tau in AICD animals after lithium treatment. D. AICD animals showed increased phosphorylation of NMDA receptors. However, Inhibiting GSK-3β normalized this increased phosphorylation of NMDA receptors in AICD-Tg animals such that they were similar to WT animals. E. Quantification of western blots for phospho-NMDAR and NMDAR. F-G. Inhibiting GSK-3β rescued impaired adult hippocampal progenitor cell (HPC) proliferation in AICD-Tg mice. HPC proliferation measured as the number of BrdU-positive cells showed increased proliferation in AICD animals after lithium treatment, which is quantified in (G). **p<0.01, ***p<0.001 one way ANOVA (Mean ± SEM). n = 5–7 mice per group. Scale bar = 50 μm.

Blocking GSK-3β using lithium resulted in decreased phosphorylated tau as detected by the phospho-tau-specific antibodies AT8 and AT180 (Fig 8A–8C), without altering total tau levels (Fig 8A). Lithium treatment also resulted in reduced somato-dendritic accumulation of phospho-tau in AICD animals (S3 Fig).

We next tested whether inhibiting GSK-3β activity reverses the increased phosphorylation of NMDAR seen in AICD-Tg mice. AICD animals on a lithium diet had decreased levels of pNMDAR compared to AICD animals on regular chow (Fig 8D and 8E). Lithium-treated animals were injected with BrdU for three days and sacrificed a day later to assess HPC proliferation. Untreated AICD-Tg mice showed a decrease in HPC proliferation (Fig 8F and 8G). Inhibiting GSK-3β activity rescued the deficit in HPC proliferation such that HPC proliferation in lithium-treated AICD-Tg mice was indistinguishable from WT animals (Fig 8F and 8G). Taken together, these results show that lithium treatment, which inhibits GSK-3β activity, can protect against tau-mediated deleterious effects in AICD-Tg mice. Moreover, our results suggest that inhibition of GSK-3β activity and lack of tau had similar effects downstream of AICD, strengthening the idea that AD-like phenotypes observed in AICD animals are mediated by the pathological accumulation of tau.

## Discussion

A growing body of evidence suggests that the toxic effects of Aβ are mediated by the microtubule binding protein tau [11, 28]. Here we show that tau is also required for the deleterious downstream effects of AICD, and contributes to AD-like pathologies in AICD-Tgmice. Our findings show that loss of tau in AICD-Tg animals protects them from impaired neurogenesis, seizure susceptibility, memory deficits, and neurodegeneration. Consistent with these observations, inhibiting the tau kinase GSK-3β activity with lithium also rescued impaired neurogenesis. By contrast, increasing the levels of tau alone results in AD-pathological features (such as impaired neurogenesis) in wild-type animals (Figs 5 and 6), which were further exacerbated in AICD;hTau double transgenic mice. Thus, these data indicate that tau plays an essential role in mediating AICD-induced AD-like pathologies *in vivo*.

**Fig 9:**
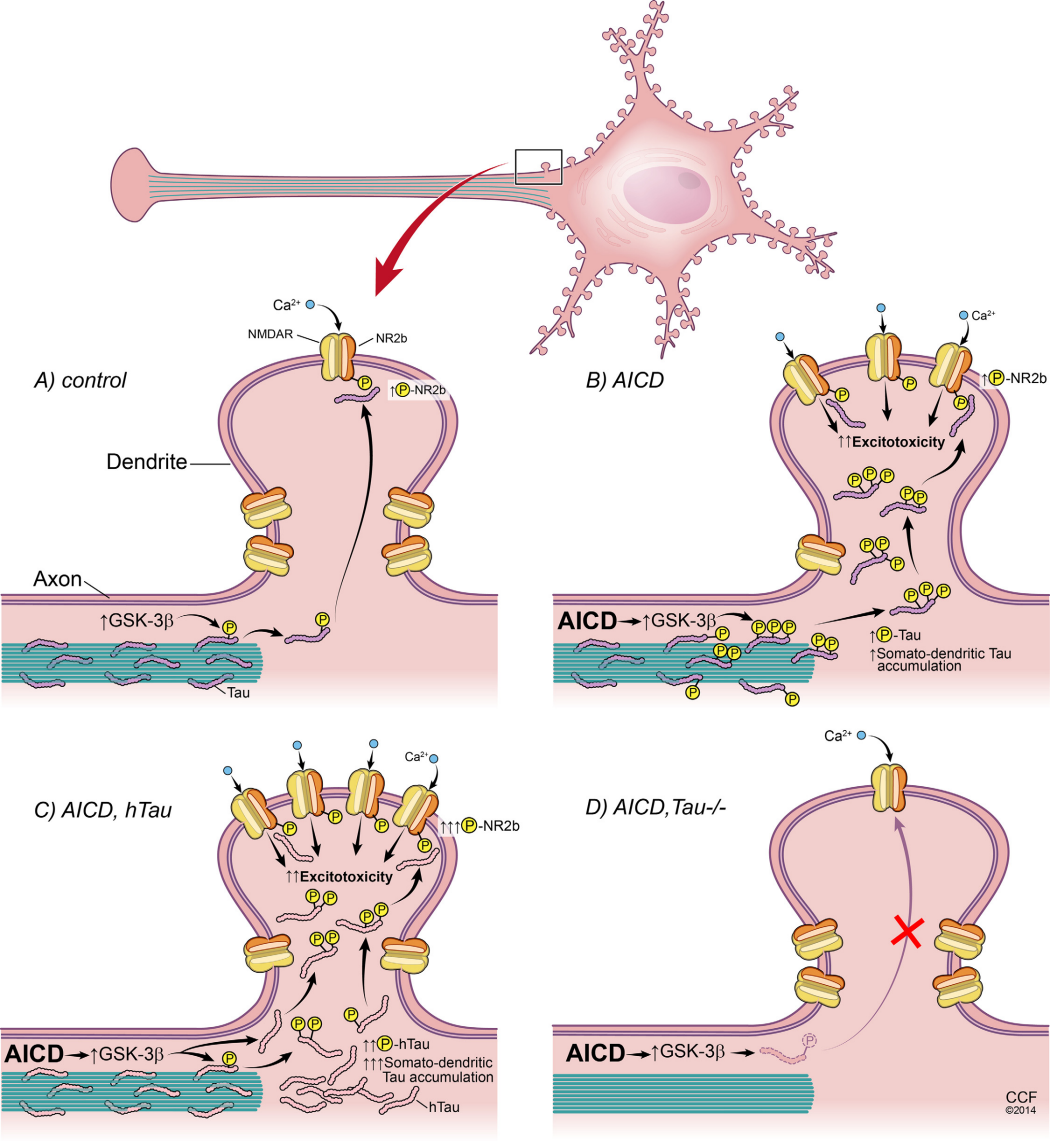
Tau mediates AICD-induced AD-like phenotypes. (A, B) AICD activates GSK-3β; this in turn phosphorylates tau, which accumulates in somato-dendritic compartments. Increased somato-dendritic tau results in a number of AD-like features in AICD animals, including phosphorylation of NMDA receptors (NMDAR), which leads to increased susceptibility to excitotoxicity. (C) Increasing the amount of tau by transgenic overexpression of human tau (h-Tau) resulted in increased somato-dendritic tau and elevated phosphorylation of NMDAR. Increased NMDAR phosphorylation led to increased susceptibility to excitotoxic effects and aggravated AD-like pathologies. (D) Loss of tau prevented increased NMDAR-phosphorylation, resulting in normalization/lowering of hyper-excitability, finally reducing/ameliorating several key AD-like features in AICD mice.

Tau is normally localized in axons where it binds and stabilizes microtubules. This binding is regulated by tau phosphorylation such that hyperphosphorylated tau fails to bind and stabilize microtubules. Hyperphosphorylated-tau is released from axons and aggregates in the soma and dendrites of neurons [39]. Such somato-dendritic accumulation of tau has been suggested to underlie the toxic effects of Aβ [28, 38, 40–42]. Interestingly, similar somato-dendritic accumulation of hyperphosphorylated tau has been shown in AICD-Tg mice [16]. GSK-3β, one of the kinases that are activated in AD [43, 44], is also activated in AICD-Tg mice [19] and leads to tau hyperphosphorylation. We propose that AICD-induced GSK-3β activation causes tau hyperphosphorylation and its accumulation in the somato-dendritic compartment, which leads to increased phosphorylation of NMDAR (Fig 9). Previous studies have suggested that dendritic tau can increase phosphorylation of NMDAR in a Fyn-dependent manner, leading to excitotoxic signaling [31, 42, 45, 46]. Thus, a similar mechanism could underlie the seizure susceptibility observed in AICD-Tg mice. Lack of endogenous tau or pharmacological inhibition of tau phosphorylation results in decreased phosphorylation of NMDAR and protects against KA-mediated excitotoxic insults in AICD transgenic mice.

Consistent with these observations, overexpression of human tau results in its accumulation in the somato-dendritic compartment, causing AD-like pathologies in WT mice. Interestingly overexpression of murine or human tau with no familial mutation can also lead to neurodegeneration [37, 47], similar to the results from our studies with human tau. Also, excitotoxic effects of Aβ resulting in neurodegeneration require tau, and were shown to require NMDAR and GSK-3β [37, 41]. Together, these findings demonstrate that tau can act as a crucial switch that regulates the toxic effects of AICD or Aβ and highlight the critical role it plays in AD pathogenesis.

Excitotoxicity can lead to a number of deleterious effects, including behavioral abnormalities and neurodegeneration. Interestingly, aging and excitotoxic stress had similar deleterious effects on AICD-Tg mice, leading to the idea that sustained occurrences of low level seizures, if untreated, can have the same effect as acute excitotoxic stimuli [18]. Lack of tau protected against this basal hyperactivity observed in young AICD-Tg mice. Absence of tau alsoprevented behavioral deficits and neurodegeneration in aged AICD-Tg mice or in young AICD-Tg mice that received KA (Fig 2). Thus, we propose that tau can mediate hyperactivity and that it also acts as a link between AICD overexpression, behavioral deficits, and neurodegeneration. Support for this concept comes from the observation that blocking GSK-3β activation with lithium reduced tau phosphorylation (present study) and proved beneficial for behavioral deficits observed in AICD-Tg mice [16]. Moreover, lack of tau proved to be protective against hippocampal neurodegeneration and learning deficits in GSK-3-overexpressing mice [48]. Though unlikely, we cannot rule out the possibility that lack of tau might alter the AICD transgene levels, and the rescue seen in AICD-Tau KO animals are due to reduced AICD transgene levels. We have earlier shown that despite the different levels of AICD in two different expressing lines (FeCγ25- high expression, FeCγ12- low expression by comparison), the resulting pathologies were similar [19] suggesting that changes in AICD levels within a certain range do not necessarily result in altered pathologies.

These studies also show that abnormal phosphorylation of tau can significantly impair adult neurogenesis. Although its functional significance remains debated, adult neurogenesis has been shown to be important in several different forms of learning and memory behaviors [25]. Moreover, adult neurogenesis is downregulated in human AD and in different mouse models of AD [27] and both AICD and Aβ have been implicated in downregulating adult hippocampal neurogenesis [23, 24, 26]. Although, the role of tau in adult neurogenesis is not clear, tau has shown to be expressed and highly phosphorylated in dentate granule cells [22, 49, 50]. Blocking dendritic accumulation of tau by inhibiting GSK-3β had a similar effect to that of the tau knockout, further supporting a role for tau in adult neurogenesis. Similar to an earlier report [49], we did not observe any significant effect of lack of tau on adult hippocampal progenitor cell proliferation and survival in WT mice. Similarly, tau knockout mice did not show any behavioral deficits, consistent with a number of previous studies [21, 29, 31]. On the other hand, increased glutamate and NMDAR activity has been shown to impair adult neurogenesis [51, 52], and could be a potential mechanism underlying impaired adult neurogenesis observed in our present study in AICD-Tg mice. Thus we provide evidence that tau controls adult neurogenesis similar to other pathological conditions seen in AD.

In conclusion, we show that tau acts as a mediator of downstream pathological events initiated by AICD, and provide compelling evidence that tau is also involved in amyloid-independent mechanisms. Reducing tau levels by therapeutic means could be an effective strategy for the treatment of AD, since tau mediates both amyloid-dependent and–independent deleterious effects. Yet caution is warranted since total loss of tau inTg-2576 mice resulted in behavioral deficits and axonal degeneration [53]. Indeed tau has emerged as the possible new target of choice for AD [54] with inhibition of tau phosphorylation and aggregation [55], reduction of tau levels by immune approaches, and microtubule stabilization [54, 56] emerging as novel therapeutic strategies. Together, our present study provides a strong rationale for the development of tau-focused therapeutics for AD.

## Supporting Information

S1 Fig AMPA receptor subtypes 1 and 2 are not perturbed by AICD overexpression either in presence or absence of tau. (A) Immunoblots of hippocampal lysates from WT, AICD-Tg and AICD;Tau^-/-^ mouse reveal no detectable change in the levels of α-amino-3-hydroxy-5-methyl-4-isoxazolepropionic acid receptor (AMPAR), the main non-NMDA glutamate receptor at the synapse. Neither GRIA1 nor GRIA2 showed any difference between the three groups. **(B)** Quantification of immunoblots for GRIA1 and GRIA2 normalized to GAPDH. N = 4–5 animals per group
(TIF)

S2 Fig Increased phosphorylation of tau after h-Tau overexpression in wildtype and AICD-Tg mice. (A) Hippocampal brain lysates from WT and AICD mouse with mouse tau or overexpressing human tau (hTau) at 3 months were western blotted using AT8 and AT180 for phospho-tau and Tau5 for total tau. hTau animals had relatively higher amounts of phospho-tau normalized to total tau amounts. **(B)** Quantification of immunoblot intensity for AT8 and AT180 normalized to Tau5. n = 5–6 for different groups.
(TIF)

S3 Fig Inhibiting GSK-3β reduces somato-dendritic accumulation of phosphorylated tau. **(A-B)** AICD transgenic mice at 3–4 mo of age have accumulation of phosphorylated tau (AT8 antibody) in the cell bodies of several neurons in the hilus (H; asterisk in **A**) and in the CA3 region **(B)** of the hippocampus. Inhibition of GSK-3β by feeding the mice lithium chow (AICD+Li) reduces the somato-dendritic accumulation of tau as detected by AT8 immunoreactivity (arrows) in the hippocampus compared to age-matched AICD-Tg mice. n = 5 for all groups. Scale bar = 100 μm for all.
(TIF)

S4 Fig Seizure progression after kainic acid injection is rescued by tau knockout. Seizure severity levels are presented for (A) 30 minutes, (B) 40 minutes, (C) 50 minutes and (D) 60 minutes after kainic acid injection. Seizure severity observed in AICD-Tg mice is rescued by Tau knockout. p values (one-way ANOVA, Mean ± SEM) are as follows: 30 min: p = 0.08; 40 min: p = 0.0049; 50 min: p = 0.0107; 60 min: p = 0.0053. n = 4–5 mice per group
(TIF)
