# Supplementary figures and images for: Tau Protein Mediates APP Intracellular Domain (AICD)-Induced Alzheimer’s-Like Pathological Features in Mice
Source: PLoS One. 2016 Jul 26;11(7):e0159435. doi: 10.1371/journal.pone.0159435 (PMC4961442; doi:10.1371/journal.pone.0159435)

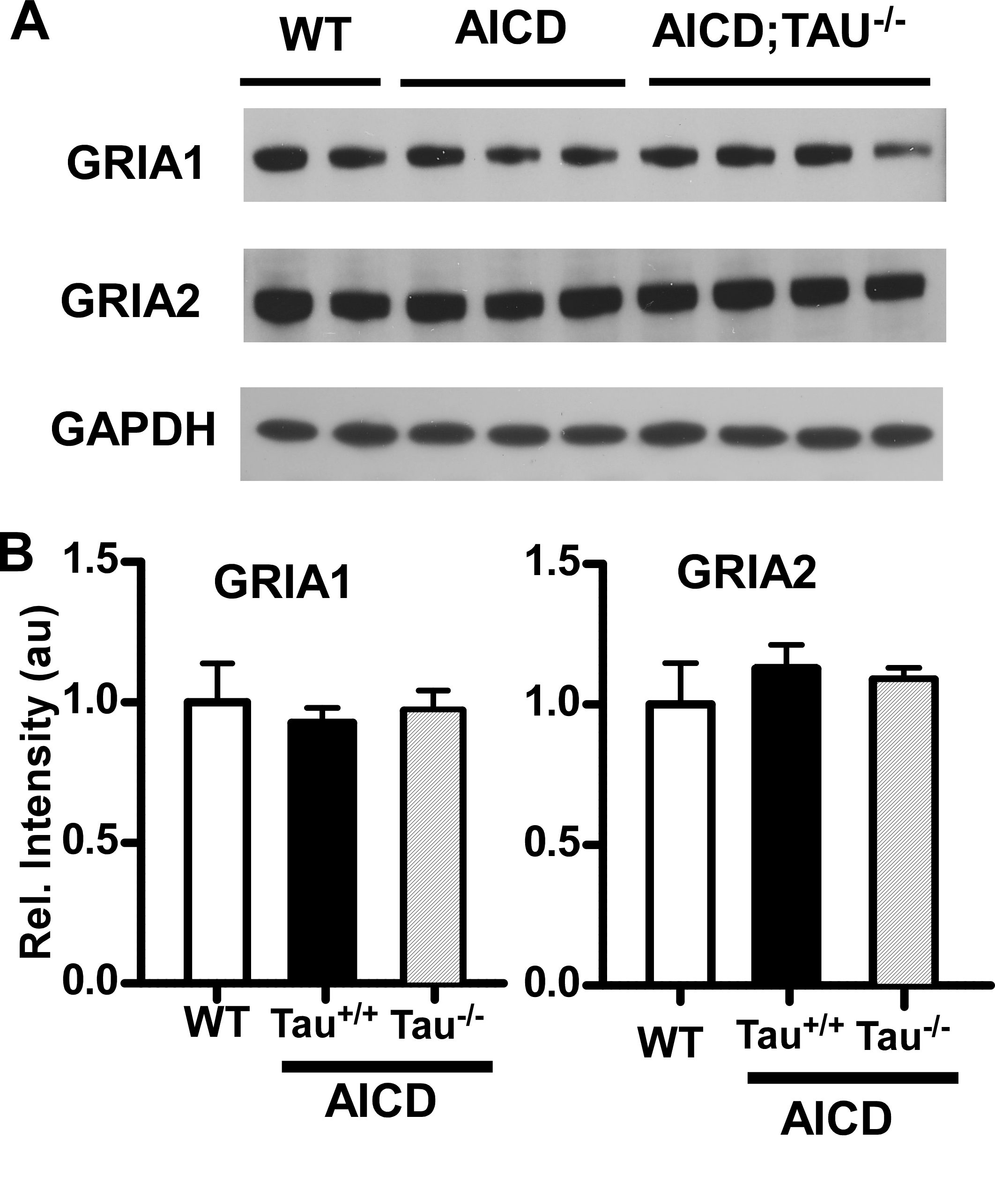

Supplement: S1 Fig — (A) Immunoblots of hippocampal lysates from WT, AICD-Tg and AICD;Tau-/- mouse reveal no detectable change in the levels of α-amino-3-hydroxy-5-methyl-4-isoxazolepropionic acid receptor (AMPAR), the main non-NMDA glutamate receptor at the synapse. Neither GRIA1 nor GRIA2 showed any difference between the three groups. (B) Quantification of immunoblots for GRIA1 and GRIA2 normalized to GAPDH. N = 4–5 animals per group (TIF) [file pone.0159435.s001.tif]

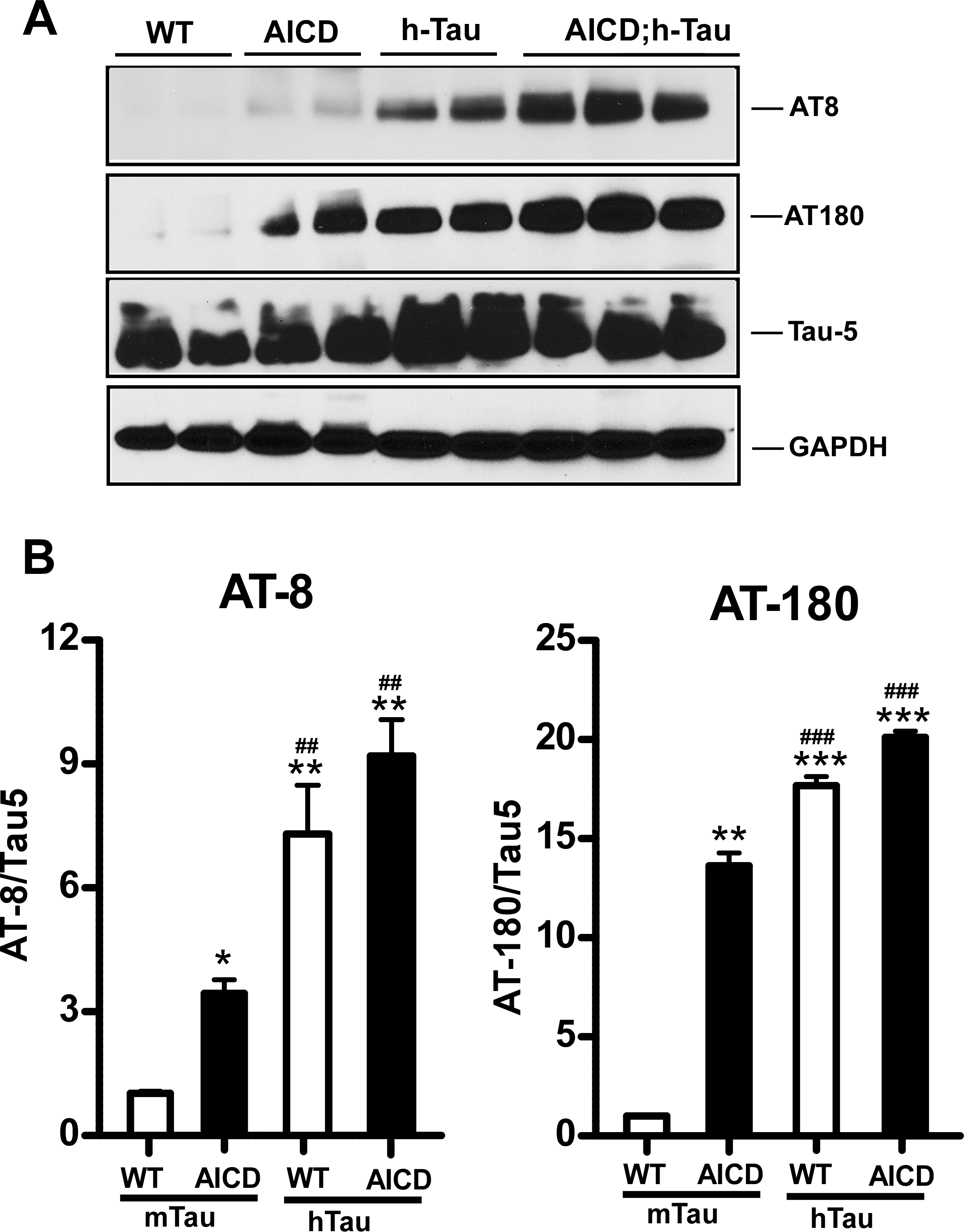

Supplement: S2 Fig — (A) Hippocampal brain lysates from WT and AICD mouse with mouse tau or overexpressing human tau (hTau) at 3 months were western blotted using AT8 and AT180 for phospho-tau and Tau5 for total tau. hTau animals had relatively higher amounts of phospho-tau normalized to total tau amounts. (B) Quantification of immunoblot intensity for AT8 and AT180 normalized to Tau5. n = 5–6 for different groups. (TIF) [file pone.0159435.s002.tif]

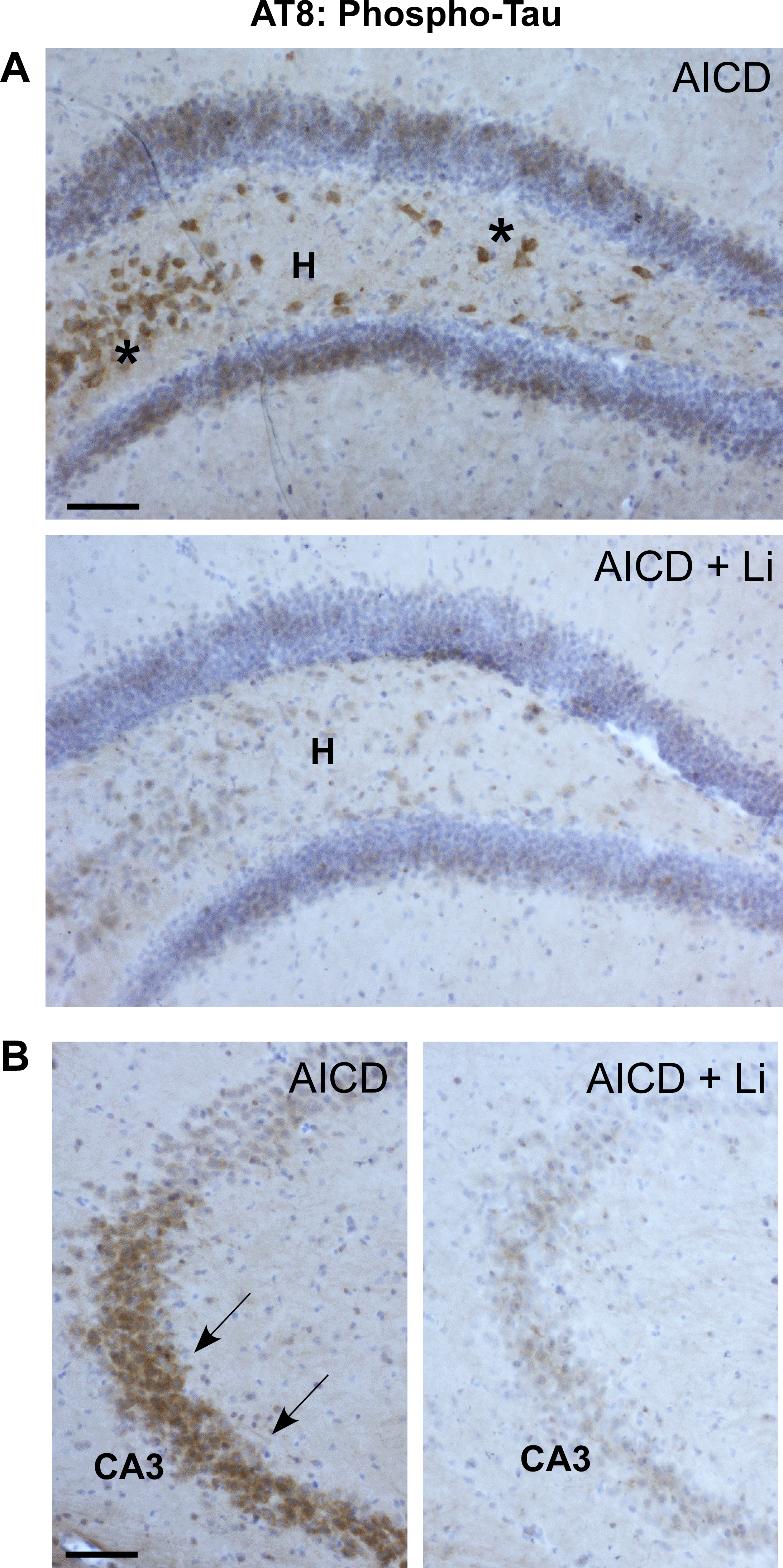

Supplement: S3 Fig — (A-B) AICD transgenic mice at 3–4 mo of age have accumulation of phosphorylated tau (AT8 antibody) in the cell bodies of several neurons in the hilus (H; asterisk in A) and in the CA3 region (B) of the hippocampus. Inhibition of GSK-3β by feeding the mice lithium chow (AICD+Li) reduces the somato-dendritic accumulation of tau as detected by AT8 immunoreactivity (arrows) in the hippocampus compared to age-matched AICD-Tg mice. n = 5 for all groups. Scale bar = 100 μm for all. (TIF) [file pone.0159435.s003.tif]

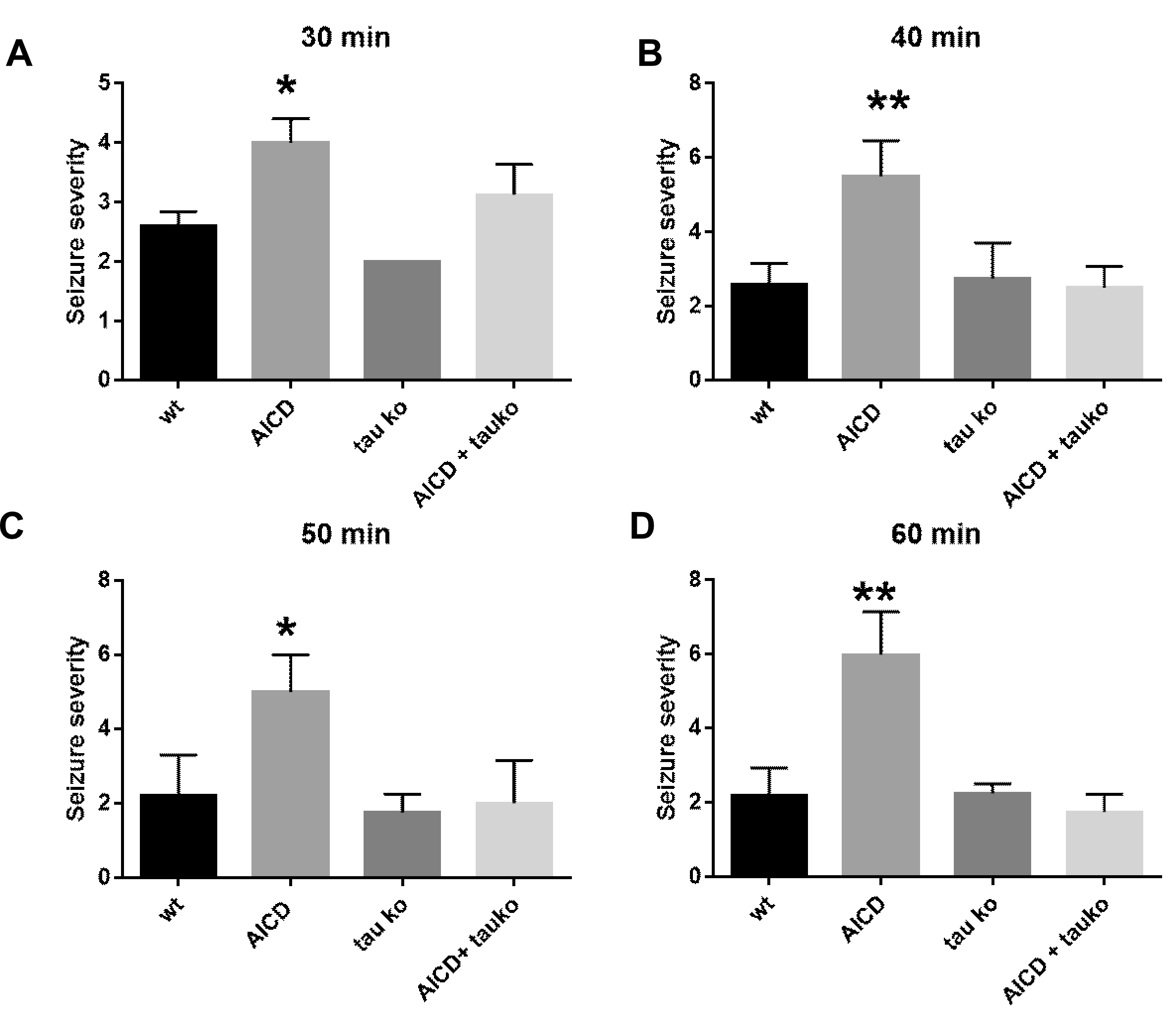

Supplement: S4 Fig — Seizure severity levels are presented for (A) 30 minutes, (B) 40 minutes, (C) 50 minutes and (D) 60 minutes after kainic acid injection. Seizure severity observed in AICD-Tg mice is rescued by Tau knockout. p values (one-way ANOVA, Mean ± SEM) are as follows: 30 min: p = 0.08; 40 min: p = 0.0049; 50 min: p = 0.0107; 60 min: p = 0.0053. n = 4–5 mice per group (TIF) [file pone.0159435.s004.tif]
